# Selective His-bundle Pacing May Preserve Intrinsic Repolarization as Well as Depolarization

**DOI:** 10.19102/icrm.2017.080301

**Published:** 2017-03-15

**Authors:** Jason S. Chinitz, Alan Scheinbach, Lawrence Ong, Kent Stephenson

**Affiliations:** ^1^Southside Hospital, Northwell Health Physician Partners, Bay Shore, NY

**Keywords:** Cardiac resynchronization, His-bundle pacing, physiologic conduction, repolarization, selective His-bundle pacing

## Abstract

A 79-year-old man with chronic atrial fibrillation underwent single-chamber His-bundle pacemaker implantation. The post-implant electrocardiogram (ECG) demonstrated selective His-bundle capture, with a narrow paced QRS and repolarization pattern similar to that of the baseline ECG. Furthermore, repolarization changes prototypic of ventricular pacing did not occur with selective His-bundle capture. While His-bundle pacing, with or without selective His-bundle capture, can preserve physiologic patterns of depolarization, only His-bundle selective pacing can preserve intrinsic ST- and T-wave patterns. Thus, the maintenance of physiologic repolarization may have various advantages, including accurate interpretation of ECG changes that are not generally interpretable in the setting of ventricular pacing.

## Case presentation

**Figure 1:**
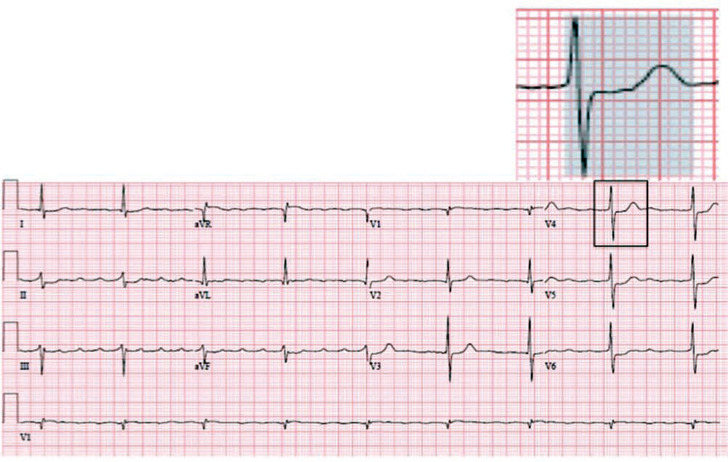
A baseline electrocardiogram. In the highlighted section, the blue background marks native QRS and ST-T wave morphology.

A 79-year-old man with chronic atrial fibrillation (AF) presented with progressive exertional dyspnea. On physical examination, he was found to be bradycardic, and demonstrated signs of mild congestive heart failure. Outpatient Holter monitoring revealed the presence of persistent AF, with slow ventricular rates and pauses up to 7.4 s. Additionally, an echocardiogram demonstrated normal biventricular function, with severe left atrial enlargement, and a 12-lead electrocardiogram (ECG) revealed atrial tachycardia with a slow ventricular rate and a narrow QRS with chronic ST abnormalities **(Figure 1)**. Owing to the anticipation of a high burden of ventricular pacing, His-bundle pacing was considered; the patient was subsequently referred for single-chamber pacemaker implantation.

A single-chamber His-bundle pacemaker was implanted using a SelectSecure™ Lead Model 3830 (Medtronic, Minneapolis, MN, USA), delivered via a C315HIS fixed shape sheath catheter (Medtronic, Minneapolis, MN, USA). During lead implantation, the lead was fixated after a His-bundle electrogram was observed on the ventricular sensing electrode, and following pacing’s production of a narrow QRS morphology and latency period from pacing to QRS complex.

**Figure 2:**
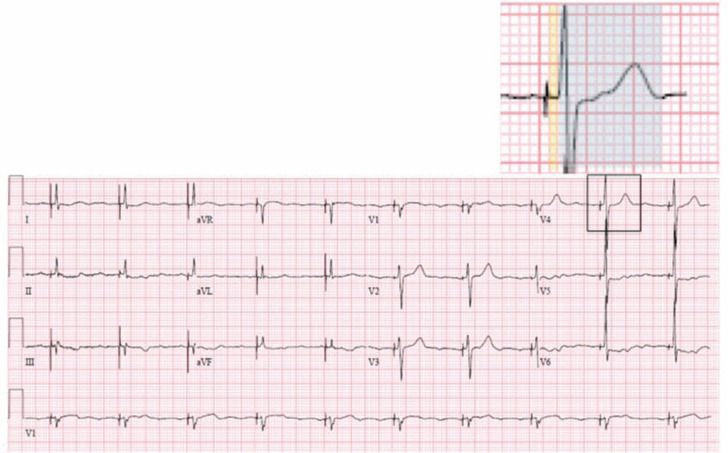
A post-implant electrocardiogram demonstrating selective His-bundle pacing. In the highlighted section, the blue background marks paced QRS and ST-T wave morphology, and the yellow background highlights the isoelectric HV interval.

**Figure 3:**
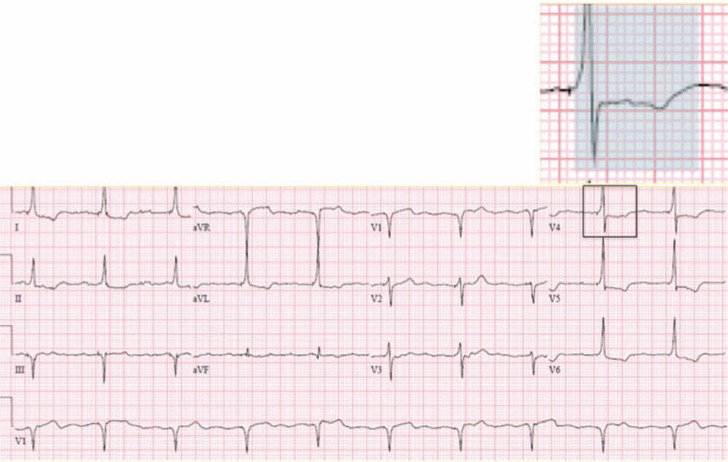
A post-implant electrocardiogram demonstrating non-selective His-bundle pacing. In the highlighted section, the blue background denotes the paced QRS with an initial slurred upstroke, indicating local myocardial capture and pacing-induced ST depression and T-wave inversion.

The patient’s post-implant ECG demonstrates ventricular pacing, with a paced QRS and repolarization pattern similar to that of the baseline ECG; the isoelectric interval from pacing spike to QRS indicates selective His bundle capture **(Figure 2)**. At higher pacing outputs, non-selective His-bundle pacing occurred, together with a pseudo-delta wave indicating fusion between His-bundle and local myocardial capture. Secondary ST-depression and T-wave abnormalities were also present **(Figure 3)**. Notably, the repolarization changes that can be typically seen with ventricular pacing were not present when selective His-bundle was achieved. During early clinical follow-up, a high degree of ventricular pacing with selective His-bundle capture was noted, and the patient reported improvement in both his symptoms and functional capacity.

## Discussion

His-bundle permanent pacing has been promoted as a means to preserve physiologic conduction with ventricular pacing, with the potential to avoid pacing-induced dyssynchrony and ventricular dysfunction.^1^ Permanent His-bundle pacing has been associated with improvements in exercise capacity, left ventricular ejection fraction, and heart failure hospitalizations, as compared with right ventricular pacing.^2,3^ Early data have suggested that either selective or non-selective His-bundle capture may have a beneficial effect with respect to cardiac resynchronization.^4^ However, in addition to maintaining physiologic depolarization pathways, selective His-bundle pacing may also preserve physiologic repolarization and intrinsic ST and T-wave patterns. In contrast, when depolarization is initiated in myocardial tissue (even in the case of non-selective His-bundle capture), abnormal repolarization patterns inevitably follow. Maintenance of physiologic repolarization may have antiarrhythmic benefits, and may even permit the accurate interpretation of acute ECG changes, such as ischemia or QT abnormalities, which are not generally interpretable in the setting of paced QRS complexes.^5,6^
